# Spider Venom: Components, Modes of Action, and Novel Strategies in Transcriptomic and Proteomic Analyses

**DOI:** 10.3390/toxins11100611

**Published:** 2019-10-22

**Authors:** Nicolas Langenegger, Wolfgang Nentwig, Lucia Kuhn-Nentwig

**Affiliations:** Institute of Ecology and Evolution, University of Bern, Baltzerstrasse 6, CH-3012 Bern, Switzerland; wolfgang.nentwig@iee.unibe.ch

**Keywords:** neurotoxins, proteomics, venomics, transcriptomics, mass spectrometry, bioinformatics, Araneae, spiders

## Abstract

This review gives an overview on the development of research on spider venoms with a focus on structure and function of venom components and techniques of analysis. Major venom component groups are small molecular mass compounds, antimicrobial (also called cytolytic, or cationic) peptides (only in some spider families), cysteine-rich (neurotoxic) peptides, and enzymes and proteins. Cysteine-rich peptides are reviewed with respect to various structural motifs, their targets (ion channels, membrane receptors), nomenclature, and molecular binding. We further describe the latest findings concerning the maturation of antimicrobial, and cysteine-rich peptides that are in most known cases expressed as propeptide-containing precursors. Today, venom research, increasingly employs transcriptomic and mass spectrometric techniques. Pros and cons of venom gland transcriptome analysis with Sanger, 454, and Illumina sequencing are discussed and an overview on so far published transcriptome studies is given. In this respect, we also discuss the only recently described cross contamination arising from multiplexing in Illumina sequencing and its possible impacts on venom studies. High throughput mass spectrometric analysis of venom proteomes (bottom-up, top-down) are reviewed.

## 1. Introduction

Many animal lineages independently developed toxic secretions. Such secretions can be either deployed through direct contact or ingestion (poison), or through a wound by stingers, teeth, fangs, harpoons, or other specialized tools (venoms) [1]. While poison is predominantly used as chemical deterrent to predation, venom is used to fight competitors or aggressors, and/or for predation. Possession of venom can thereby provide a possibility to subdue a prey without getting involved in a potentially dangerous fight [1]. Venoms contain dozens of different toxins, many of which acting as neurotoxins by targeting ion channels and receptors at membranes of excitable cells [2]. Some venomous animals pose a serious threat to primates. Among them, snakes have probably been the first predators of higher mammals (Placentalia) and have promoted the development of visual systems and fear in primates [3,4]. Potential threats to primates is also seen as the main reason for the long-lasting fascination of humans towards venomous animals [5].

Already long time ago, humans started to use animal venoms for their benefit. Native Americans, for example, applied rattle-snake venom to the tips of their arrows to increase the damage caused by a strake [6], and in Ayurveda, a historical Indian medicine, cobra venom was used to treat arthritis [7]. Today, more than ever, venoms are recognized as rich source for development of pharmaceuticals and venom research is mainly driven by screenings for possible drug leads [8]. Multiple drugs have been developed from venoms. Among them are an analgesic from a cone snail neurotoxin [9] and an antihypertensive from a snake venom angiotensin-converting-enzyme inhibitor [10]. Venom components are also increasingly used in research of their biological targets (e.g., ion channels) [11], and venoms of imminent danger to humans are studied for development of envenoming therapies [12]. Also, venoms of animals preying on insects are studied for development of insecticides [13].

Besides snakes and some marine animals, commonly known venomous animals especially comprise arthropods, such as scorpions, various insects, and spiders. Spiders are after insects the largest taxonomic group of terrestrial organisms and occupy most ecological niches of our planet [14]. Spider venom has for a long time received only small attention due to its limited impact on human health. Research only gained in focus after realizing the huge pharmaceutical potential of spider venom peptides [15].

After a long period dominated by Edman degradation, new DNA sequencing techniques and soft ionization techniques for mass spectrometry (matrix-assisted laser desorption/ionization (MALDI), and electrospray ionization (ESI)) accelerated also spider venom research. Meanwhile, dozens of venom gland cDNA libraries, sequenced by Sanger, Roche 454 and Illumina techniques [16,17,18,19,20,21,22,23,24] diversified our knowledge on spider venom within less than two decades considerably. Here we review the state of the art of the composition of spider venoms and the currently available methods to analyze it.

## 2. Spider Venom Composition

### 2.1. Spider Venom

Venom is produced in specialized venom glands. Such glands are already present in the oldest taxon of spiders, the Mesothelae, which developed about 300 million years ago [25,26]. During the evolution of spiders, the venom glands and venom delivery systems evolved from the very small and hardly functional glands of Mesothelae to well-developed glands located in the basal cheliceral segment of mygalomorph spiders (“tarantulas”). With the separation and evolvement of araneomorph spiders (“true spiders”), the venom glands relocated into the prosoma, where they could occupy more space, and gained in size [14]. Together with the relocation of the venom gland, also the orientation of the chelicerae changed from orthognath (fangs face straight down) in mygalomorph spiders to labidognath (fangs directed towards each other) in araneomorph spiders [27]. This change is reflected in the older name for these spider groups, Orthognatha and Labidognatha, respectively. The relocation of the venom gland and reorientation of the chelicerae enabled a reduction in body size without major impact on a spider’s defensive and attacking power. The reduction in body size was a prerequisite for the use of functional webs, especially aerial webs, which have developed in several lineages. Today over 90% of all known spiders are araneomorph and among them 45–50% build webs [14].

Independently from their ability to build a web or not, the use of venom for hunting and defense seems to be a story of success for spiders. Loss or reduction of venom glands is only described for spiders of the family Uloboridae, which instead intensively wrap their prey in cribellate silk [28]. Not a loss, but a partial refunctionalization of the venom gland is observed for spitting spiders (Scytodidae). In addition to venom, their venom glands produce a protein-based glue that is spit onto a prey from a distance of approx. 2 cm and leads to immediate immobilization [29].

Most spiders are polyphagous and naturally prey on invertebrates, mainly insects [14]. Specialization on one type of prey is rare and restricted to a few spider taxa: specialization on ants has been reported for many *Zodarion* species (Zodariidae) [30], *Ammoxenus* species (Ammoxenidae) feed exclusively on termites [31], *Ero* species and other mimetids (Mimetidae) as well as *Portia* species (Salticidae) prey only on spiders, spiders of the genera *Celaenia* and *Mastophora* (Araneidae) are highly specialized moth predators [32], and *Bagheera kiplingi* (Salticidae) is the only spider that predominantly feeds on plants [33]. To what extend a specialization on one type of prey affects the composition of the venom is largely uninvestigated in spiders. However, a certain heterogeneity between venoms of individuals of the same species depending on age, gender, or geographic location has been reported and supports a possible adaptation of the venom composition [34,35]. Adaptive evolution of venom components on prey is described for other venomous animals, such as snakes [36,37,38], and cone snails [39,40].

Spider venom is efficient against a broad spectrum of prey groups, its effect on humans, however, is largely overestimated. Bites with serious effects are limited to a few spider taxa only, including the mygalomorph Australian funnel-web spiders (*Atrax* sp. and *Hadronyche* sp., Atracidae), and the araneomorph recluse spiders (*Loxosceles* sp., Sicariidae), widow spiders (*Latrodectus* sp., Theridiidae), and armed spiders (*Phoneutria* sp., Ctenidae) [41]. The low impact on humans is also reflected in the number of recorded deaths per year. This is estimated to be less than 5 deaths per year worldwide [42]. Comparatively, 180 fatalities were recorded from scorpion stings [43] and over 125,000 fatalities per year were reported for snake bites [44]. Reasons for the low number of spider related fatalities in humans are that (1) >85% of all spider species are <15 mm body length and produce only a very small quantity of venom (usually <10 µL per pair of venom glands), (2) most taxa are not aggressive, (3) most spiders do not have mouthparts powerful enough or chelicerae long enough to penetrate the human skin [45], and most importantly, (4) humans are no prey for spiders, thus, evolutionary distant from a typical spiders’ prey, which the venom is optimized on. 

The toxicity on humans is also directly influencing the research on venom. The knowledge on venom components was for a long time largely restricted to spiders with medical importance to humans or to spiders with relatively large body sizes, making them easy to handle. The ArachnoServer [46,47], a spider specific toxin database, only lists 1561 entries from 100 species (as of 15 May 2019), while 48,389 spider species are described to exist in the World Spider Catalogue [48]. This relatively low rate of knowledge, in combination with the high diversity in knowledge-depth on single venoms or venom component groups, makes it challenging to provide a comprehensive overview on spider venom. 

In general, the spider venom of a given species is a mixture of over hundred components acting on different targets including various receptors, mostly located in the muscular or nervous system, cell membranes, and extracellular matrix. Although single components may be toxic, it is the synergistic action between the components, which deploys the full toxicity of the venom. The peptide and protein concentration of spider venom is often relatively high with reports reaching from 65 µg/µL [49] to 150 µg/µL [50]. It is evident, that production of a fluid with these amounts of peptides/proteins comes at high energetic costs for the spider. Hereof some spiders have been shown to economically use their venom by adaptation of the injected amounts depending on prey size and movement [51] or endangerment by the prey [52], a venom usage strategy described by the venom optimization hypothesis [53].

Spiders produce their venom components in specialized secretory cells in the venom gland. The venom gland is surrounded by muscular layers controlling venom release by squeezing the venom gland [54]. Depending on the spider species, venom is released into the glandular lumen by disintegration of entire cells (holocrine secretion) or by pinch off of parts of cells to form extracellular membrane-bound vesicles and release of venom components from these vesicles (apocrine secretion) [54,55,56].

Spider venom components are typically divided into four groups. (1) Small molecular mass compounds (SMMSs), (2) antimicrobial peptides (only a few spider families), (3) peptide neurotoxins, and (4) proteins and enzymes. Details on the different groups of venom components are given in the following sections.

### 2.2. Small Molecular Mass Compounds

Small molecular mass compounds (SMMCs) are thought to be present in most spider venoms. They include ions, organic acids, nucleotides, nucleosides, amino acids, amines, and polyamines [14]. Many SMMCs are only marginally investigated compared to venom peptides and systematic studies are rare. The here presented data and many generally valid conclusions on spider venom SMMCs are mostly derived from only a few investigations of venoms of selected species.

Venoms of the spider *Cupiennius salei* (classically listed as Ctenidae, recently moved to Trechaleidae) and *Aphonopelma hentzi* (published as *Eurypelma californicum*) (Theraphosidae) are rich in potassium (approx. 200 mM, and 70 mM, respectively) and poor in sodium (approx. 10 mM) [50,57]. These cation concentrations are opposite to the hemolymph concentrations. The high potassium content of venom is described to induce depolarization of excitable cell membranes, leading to paralysis of the prey, and to synergistically enhance the activity of venom peptides [58,59].

Spider venom has an acidic pH with pH values reported between 5.3 and 6.1. Main contributors to this acidic environment are organic acids, primarily citric acid, which is by far the most described organic acid present in spider venom [14]. Citric acid is also present in snake, scorpion, bee, wasp, and ant venoms and was proposed to reversibly inhibit divalent cation dependent enzymes (e.g., phospholipase A2 or metallopeptidases) in the venom gland by complexation of Ca^2+^ and Zn^2+^. After venom injection, dilution of citric acid would shift the equilibrium in direction of complexation of the cations by the venom enzymes. The enzymes would thereby get reactivated [60].

Many SMMCs effect neuronal or neuromuscular signal transduction. Nucleosides (some with sulfate-ester) are known from many spider venoms and have been reported to block kininate receptors and L-type Ca^2+^ channels [61]. Nucleosides are a major component of the venom of *Loxosceles* sp. [62] and they even make up to 50% of the venom dry weight in *Tegenaria* sp. [63].

Acylpolyamines are a diverse class of molecules built from an aromatic acyl group and a polyamine backbone (some with amino acids in the backbone). They possess masses ranging from 350 to 1000 Da and are described to plug open ionotropic glutamate receptors and to induce paralysis in a prey item [42,64]. Amino acid-containing acylpolyamines are described for Araneidae, non-amino acid-containing for Agelenidae, Amaurobiidae, and Pisauridae (all araneomorph) and Ctenizidae, Theraphosidae, and Atracidae (mygalomorph). Glutamate receptors are also blocked by polyamines not containing an acyl group (e.g., spermidine, spermine) reported from the venom of some spiders [65]. Other SMMCs reported form spider venom act as neurotransmitter in invertebrates. Among them GABA, glutamate, acetylcholine, adrenaline, and biogenic amines, such as noradrenaline, histamine, octopamine, tyramine, serotonin, and dopamine [14,42,66,67]. Further SMMCs include all proteinogenic amino acids, the non-proteinogenic amino acid taurine, and nucleotides [14].

### 2.3. Antimicrobial Peptides

**Figure 1:**
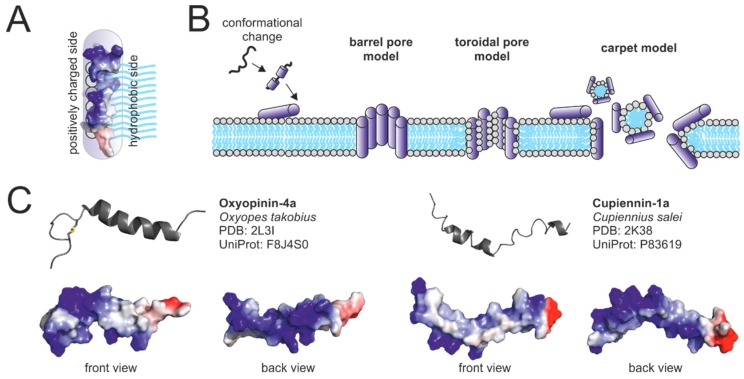
Antimicrobial peptides (AMPs) and their proposed mechanism of action. (**A**) Model of interaction between an AMP and phospholipids. AMPs assume an amphipathic α-helical structure in proximity to cellular membranes. The hydrophobic side of the helix (white) inserts into the membrane and interacts with the phospholipid side chains. The positively charged side (blue) interacts with negatively charged lipid head groups. (**B**) Models of membranolytic actions of AMPs. (**C**) NMR-based 3D structures of two antimicrobial peptides from spider venom. Electrostatics were computed using PDB2PQR [70]. Blue surfaces represent positively charged surfaces; red negative charged; and white neutral.

Antimicrobial peptides (AMPs) are also termed cytolytic or cationic peptides. AMPs often feature high positive net charges and a high number of hydrophobic amino acids. They are widely distributed as major components of animal immune systems and are also present in various arthropod venoms, such as ant, scorpion, bee, and wasp venoms [68,69]. AMPs disrupt the integrity of cellular membranes. They are attracted to the surface of cellular membranes owing to interaction of their positively charged amino acid side chains with negatively charged head groups of phospholipids or other negatively charged surface molecules. In proximity to membranes, AMPs exhibit an amphipathic α-helical structure. This amphipathic structure facilitates the insertion of AMPs into the cell membrane. Positively charged parts of the α-helix interact with negatively charged phospholipid head groups and hydrophobic parts of the α-helix with phospholipid tails (Figure 1A).

**Figure 2:**
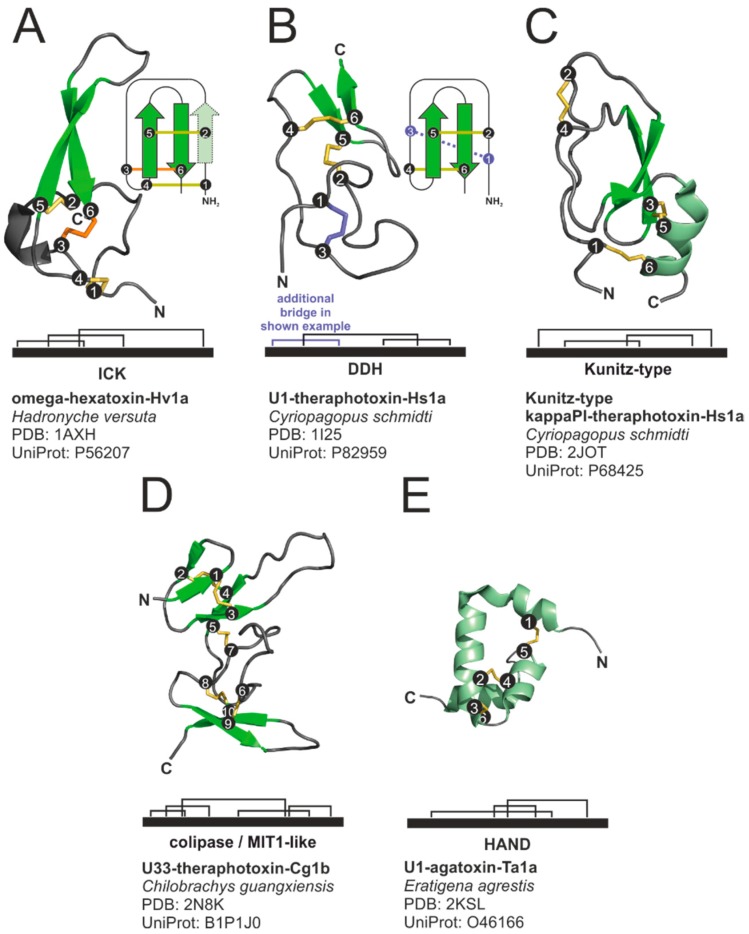
Structural motifs of cysteine-rich spider venom peptides. Disulphide connected cysteines are numbered and disulphide bridges are shown in yellow. A linear schematic representation of the disulphide bridge pattern is shown below the corresponding 3D structure. (**A**) Inhibitor cystine knot motif. The disulphide bridge colored in orange penetrates the ring opened by the peptide backbone and the two other disulphide bridges. (**B**) Disulphide-directed β-hairpin motif. The disulphide bridge colored in blue is optional for this motif. (**C**) Kunitz-type motif. (**D**) Colipase or MIT1-like motif. (**E**) Helical arthropod-neuropeptide-derived motif.

Multiple models for the membranolytic activity of AMPs have been proposed (Figure 2B). In the barrel pore model, multiple AMPs oligomerize and insert perpendicularly into the membrane, forming a pore where the hydrophilic region of the α-helices face the center of the pore. The toroidal pore model is similar to the barrel pore model but assumes that pore-forming AMPs always interact with lipid head groups. In the carpet model, AMPs coat parts of membranes leading to peptide-micelle formation and formation of holes in the membrane [71]. The efficiency of the membranolytic effect may be influenced by the lipid composition of cellular membranes and the membrane potential. Cupiennins (AMPs from the venom of the spider *Cupiennius salei*) have been reported to strongly interact with negatively charged 1-palmitoyl-2-oleoyl phosphatidyl-DL-glycerol vesicles, but only weakly with zwitterionic 1-Palmitoyl-2-oleoyl phosphatidylcholine vesicles [72]. Oxyopinins (from *Oxyopes takobius*, erroneously identified as *O. kitabensis*) more efficiently lyse phosphatidylcholine (zwitterionic) vesicles than phosphatidylethanolamine (zwitterionic) or phosphatidic acid (negative) vesicles [73]. Phospholipid headgroup size and related membrane density and fluidity have also been named as possible influencers of the cytolytic efficiency [73]. In addition, latracins (*Lachesana tarabaevi*) only act on membranes with a negative potential, resembling the potential of living cells (>−100 mV), but not on membranes with a positive potential [74]. The efficiency of the cytolytic activity may also be influenced by molecules exposed on the surface of membranes, such as sialic acid, which was shown to promote binding and hemolytic activity of a cupiennin 1a analogue (*Cupiennius salei*) [75].

AMPs have so far only been reported to occur in venoms of *Cupiennius salei* and the spider families Zodariidae, Lycosidae, and Oxyopidae [14]. Spider venom AMPs feature masses between 2–5 kDa and are, in most cases, cysteine free peptides. It is not unusual, that they contain a glycine or proline residue, inducing a hinge in their α-helical structure. Two representatives of spider venom AMPs are shown in Figure 1C. 

Some spider venom AMPs are supposed to act according to the toroidal pore model [76,77,78], others according to the carpet model [74,79] (Figure 1B). In addition, Corzo et al. [73] hypothesized, that the mechanism of action may depend on the type (zwitterionic or anionic) of lipids, the target membrane is composed of. Besides the membranolytic activity of AMPs, activities including the inhibition of the neuronal nitric oxide synthase [80] and of the superoxide producing NADPH oxidase [75] have been reported for single peptides. Other hypothesized functions include antimicrobial defense of gland and delivery system [69], or, controversially discussed, the pre-digestion of prey [14,81]. AMPs of the spiders *Cupiennius salei* and *Oxyopes takobius* have been shown to synergistically act with neurotoxins [52,58,73]. It has been proposed that AMPs facilitate the movement and access of neurotoxins to their targets [58]. The exact mechanism of the synergistic action, however, remains uninvestigated.

### 2.4. Cysteine-Rich Peptides

Cysteine-rich peptides are the best investigated venom components and are believed to exist in most spider venoms [14]. Spider venoms typically contain dozens of different cysteine-rich peptides, whereof most are thought to act on channels and receptors on membranes of excitable cells. That is why they are often referred to as neurotoxic peptides or neurotoxin-like peptides. Cysteine-rich peptides typically have molecular masses between 3 and 9 kDa, and feature ≥6 cysteine residues in conserved structural motifs [14].

#### 2.4.1. Structural Motifs

The most prominent structural motif of spider venom cysteine-rich peptides is the inhibitor cystine knot (ICK) motif. A number of other structural motifs are less frequently found in spider venom peptides. These are (1) the disulphide-directed β-hairpin (DDH) fold, (2) the Kunitz motif, (3) the colipase like fold, also known as MIT1-like fold, and (4) the helical arthropod-neuropeptide-derived (HAND) motif.

The ICK motif was initially characterized by a triple stranded antiparallel β-sheet stabilized by six cysteines arranged in the disulphide bonds C1–C4, C2–C5, C3–C6. The cystine C3–C6 thereby penetrates the characteristic ring formed by the two other disulphide bonds and the peptide backbone (Figure 2A). This cystine structure is often referred to as cystine knot, and peptides featuring this knot as knottins [82,83]. Meanwhile, some ICK-like peptides have been found to lack the first β-sheet, but still featuring the β-hairpin formed from the β-sheets 2 and 3 [84]. Many peptides feature the ICK as core motif but contain additional cysteines. A common variation is the inclusion of a fourth disulphide bridge in the loop of the β-hairpin between C5 and C6 of the simple ICK motif (e.g., [85]). Peptides featuring the ICK motif are found in the venom of various organisms besides spiders (e.g., scorpions [86], and cone snails [87]), but also in peptides with different functions, such as protease inhibitors of plants [88], an immunopeptide from horseshoe crabs [89], or an elicitor protein of a fungal tomato pathogen [90].

The DDH fold (Figure 2B) is widely distributed among different organisms [84]. It features two mandatory disulphide bonds (C1–C3, C2–C4) and an antiparallel β-hairpin, comparable with the β-hairpin formed by the beta sheets 2 and 3 of ICK peptides [14]. The loop before the first β-sheet (between Cys 2 and Cys 3) commonly comprises a Gly or Pro residue supporting a tight turn of the peptide backbone [91]. Some scientists considered the DDH as an ancient fold, from which the ICK fold evolved [91], others suggested, that the DDH is a derivative of the ICK [92]. The Kunitz motif (Figure 2C) is the active domain of a class of serine protease inhibitors. It usually features an antiparallel β-sheet and a C-terminal α-helical part constrained by three disulphide bridges (C1–C6, C2–C4, C3–C5). Venom peptides with this motif were originally isolated from snakes (green mamba) [93] and later also identified in the venom of other animals including cone snails [94] and sea anemones [95]. Despite their structural similarity to protease inhibitors, many Kunitz domain containing venom peptides have been described to block potassium ion channels [96,97,98]. Some only retained very weak inhibitory effects on serine proteases [98].

A colipase like fold (Figure 2D) of a venom peptide was first reported for the snake toxin mamba intestinal toxin 1 (MIT-1) [99,100]. Later, homologues were reported from frog skin [101], humans, mice [102], and further animals [103]. These peptides commonly comprise the four amino acids AVIT at their N-terminus. Accordingly, the peptide family is also known as AVIT family [104]. The characteristic disulphide bridge pattern (C1–C4, C2–C5, C3–C7, C6–C9, C8–C10) is similar to the one of colipases and the C-terminal region of Dickkopf proteins [104]. This fold has been discussed to conform two head to tail connected DDH motifs [91]. MIT-1 and its homologue from the skin of the toad *Bombina variegata* were shown to bind mammal prokinectin receptors and to cause short term gastric smooth muscle contraction and hyperalgesia [101]. Dickkopf-like activities could not be detected [104]. Homologues in spider venom have first been reported from Australian funnel-web spiders. These peptides, however lack the N-terminal AVIT domain and they do not bind prokinectin receptors, nor induce contractions of gastrointestinal smooth muscle, or cause toxic effect on house crickets [105,106]. Related peptides have meanwhile also been reported in other spiders [107], their function, however, remains unknown.

HAND toxins (Figure 2E) are composed of four tightly packed α-helices stabilized by three disulphide bridges (C1–C5, C2–C4, C3–C6). Unlike the vast majority of other spider venom peptides, their precursors do not contain a propeptide. HAND toxins are widespread in araneomorph spiders (e.g., *Nephila* sp., *Loxosceles* sp., *Sicarius* sp., *Heteropoda* sp., *Latrodectus* sp., *Tegenaria* sp.) and are also found in centipedes, wasps, ticks, and scorpions [108,109]. They are thought to have convergently evolved from neuropeptide hormones of the crustacean hyperglycaemic hormone and arthropod ion transport peptide families [109,110]. The first HAND toxins isolated from spider venom, the latrodectins, were co-purified with large pore-forming proteins (alpha-latrotoxins) and were thought to act as co-factors for them [111,112]. Latrodectins were reported to lack toxicity on mice and cockroach [113]. Today, HAND toxins are also known for many spiders, which do not contain alpha-latrotoxin homologues. Toxic effects are not reported with one exemption: U1-agatoxin-Ta1a (O46166) from the spider *Eratigena agrestis*, shows insecticidal activity probably resulting from directly targeting the insect’s central nervous system (CNS) [114].

Most commonly, spider venom cysteine-rich peptides are monomers. To the best of our knowledge, there are currently only two heterodimeric structures known (U2-ctenitoxin-Cs1a, P83919, *Cupiennius salei* [59], and omega-agatoxin-1A, P15969, *Agelenopsis aperta* [115]). These two toxins are further discussed below.

#### 2.4.2. Targets of Neurotoxic Peptides

Neurotoxic peptides modulate a broad range of channels and receptors on membranes of excitable cells (e.g., nerves and muscles). Their target specificity is thereby not reliably predictable given the structural motif or the primary sequence of a peptide. Specificities and potency are experimentally investigated, frequently in patch-clamp experiments, e.g., [116]. Neurotoxic peptides form stable complexes with channels or receptors and are effective already at nanomolar concentrations. Thus, neurotoxic peptides exhibit their toxic effects already at concentrations, which are at least one order of magnitude lower than the one of non-selective toxins, such as AMPs. The toxic effects, however, can be dependent on the type of prey, the specificity of the neurotoxins for the specific channels, and the receptors expressed by that prey [14,81]. When counting today (15 May 2019), 481 out of 1561 entries listed in the ArachnoServer have a described target. Of them 46% act on voltage-activated sodium channels (Na_V_) of different subtypes by either delaying their inactivation (e.g., delta-ctenitoxin-Pn2c, O76199), inhibiting their opening (e.g., mu-theraphotoxin-Hs2a_1, P83303), or shifting their potential limit for activation (e.g., beta-theraphotoxin-Cm1a, P84507). Approx. 37% inhibit voltage gated calcium channels (Ca_V_, e.g., omega-ctenitoxin-Cs1a, P81694), and 15% inhibit voltage gated potassium channels (K_V_, e.g., Kunitz-type kappa-PI-theraphotoxin-Hs1a, P68425). Other targets include acid-sensing ion channels (e.g., pi-theraphotoxin-Pc1a, P60514), NMDA-glutamate-receptors (e.g., gamma-ctenitoxin-Pn1a, P59367), calcium-activated potassium channels (e.g., kappa-hexatoxin-Hv1e [sic], S0F1M9), and transient receptor potential (TRP) channel (e.g., tau-theraphotoxin-Gr1a, M5AXK5). The action of a toxin is not necessarily restricted to one type of channel (e.g., beta/kappa-theraphotoxin-Hlv1a, B3EWN3; mu/omega-theraphotoxin-Hs1a(1), B3FIR8). Ion channels on membranes of excitable cells are responsible for proper signal transduction. Calcium channels are involved in neurotransmitter release from presynaptic cells, voltage gated sodium channels enable the transport of the action potential along excitable cells, and voltage gated potassium channels are crucial for restoring a resting state in depolarized cells. Disruption of the channels function (e.g., by inhibition or non-natural activation through toxins), may affects coordination, locomotion, respiration, and cardiac functions leading to various symptoms including convulsions, paralysis, and death. Toxins acting on the capsaicin receptor (transient receptor potential channel) may induce inflammatory pain [117,118].

#### 2.4.3. Nomenclature of Toxins

In 2008, King et al. [119] recognized the need of a universal nomenclature for maintaining an overview on the rising number of known peptides of venomous animals and introduced a rational nomenclature based on their molecular target. This three-part-nomenclature includes a (1) Greek letter describing a toxin’s activity or target (e.g., “ω” for Ca_V_ blockers, or “U” for unknown), followed by (2) an indicator of the source organism’s family, genus, and species, (e.g., “ctenitoxin-Cs” for *Cupiennius salei* (Ctenidae), and (3) a number to distinguish toxins with identical parts 1 and 2. Examples for the nomenclature of peptides acting on the most relevant targets for spider venoms are given in Table 1. The big advantage of this nomenclature is the fast identification of a toxin’s target in its name. This, however, is only possible, if the target is known and the ArachnoServer lists currently (15 May 2019) 61% of toxins with unknown target. Recent developments in omics techniques resulted in a flood of toxin sequences with no associated functional data, deposited in online repositories. For such toxins, the nomenclature is restricted to consecutively numbering the toxins of a spider e.g., U1-ctenitoxin-Cs1a, U2-ctenitoxin-Cs1a, U3-ctenitoxin-Cs1a.

In addition, the linkage of this nomenclature to the spider family, genus, and species names can lead to confusion caused by taxonomical rearrangements of spiders. As example, *Atrax* and *Hadronyche* species have recently been moved from Hexathelidae to an own family, Atracidae [48]. Consequently, delta-hexatoxin-Hf1a from *Hadronyche formidabilis* would require renaming to delta-atracitoxin-Hf1a. Also, U_10_-theraphotoxin-Hs1a would require renaming to U_10_-theraphotoxin-Cs1a because the theraphosid *Haplopelma schmidti* has meanwhile been moved into the genus *Cyriopagopus*.

#### 2.4.4. Molecular Binding of Neurotoxins

Binding of toxins to their receptors is often studied by combination of patch-clamp electrophysiology with recombinant expression of the toxin and/or receptor and site directed mutagenesis (e.g., alanine screening), [120,121,122,123]. Structural data of toxins (e.g., from NMR spectroscopy) are often included to study structure activity relationships (SAR). Exceptional insight is also gained from the only recently published first crystal structures of spider venom toxins bound to their target channels [124,125,126].

Most, but not all [127] spider venom neurotoxins are today thought to bind their target channels indirectly. They first partition into the cellular membrane and then laterally diffuse to reach the target channel. Hereof, several neurotoxic peptides have been found to bind model membranes and to form trimolecular complexes consisting of the toxin bound to the membrane and the target receptor [128]. Membrane contact is thought to enhance the affinity of inhibition as a large part of the binding energy deviates from membrane binding [129]. Furthermore, membrane binding was also shown to induce structural changes of a toxin [130], and cause ideal positioning of a toxin to bind voltage-gated ion channels [131].

**Figure 3:**
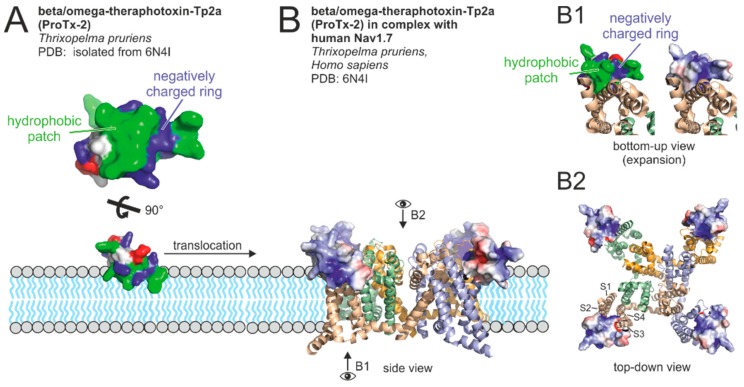
Binding of neurotoxins to a voltage gated ion channel. Peptides are shown space filling, ion channels as cartoons with every domain in a different color. (**A**) Hydrophobic patch with surrounding ring of charged amino acids. Hydrophobic amino acids are shown in green, negatively charged in blue, and positively charged in red. (**B**) Four ProTx-2 toxins bound to the human Na_V_1.7 channel in side view. Toxin surface represents surface charge. Eye-icons and arrows indicate the viewing angel in panels B1 and B2. (**B1**) View on one voltage-sensing domain of the channel bound to the toxin from the inner side of the membrane towards outwards. (**B2**) View on the toxin-channel complex form the top. Helices of the voltage sensing domain are indicated with S1–S4.

Neurotoxin–membrane interaction was extensively studied with VSTx1 (kappa-theraphotoxin-Gr3a, P60980). This tarantula venom toxin induces a thinning of the phospholipid bilayer and locates superficially at the lipid–water interface inserting several hydrophobic amino acids into the membrane interior, while basic residues mainly retain in the aqueous phase [131]. The toxin region interacting with the membrane thereby resembles a hydrophobic patch surrounded by a ring of positively charged amino acids. This motif is present in many spider toxins and is generally thought to be important for membrane partitioning (Figure 3A) [128,132,133]. Electrostatic interactions between the ring of positively charged amino acids and lipid head groups thereby seem to be important for membrane binding as some toxins were found to have a higher affinity to membranes with high amounts of negatively charged lipids (e.g., phosphatidylglycerols) than to membranes with zwitterionic lipids (e.g., phosphatidylcholines) [127,132,134,135]. The targets of most spider venom neurotoxins are voltage-gated sodium, potassium, and calcium channels. These channels are generally composed of a central pore determining the ion selectivity and four surrounding voltage-sensing domains regulating opening and closing of the central pore. While most K_V_ are tetramers, most Na_V_ and Ca_V_ are composed of one polypeptide with four repeated similar domains (1 to 4). Every repeat (or subunit of the K_V_ channel) contains six transmembrane helices (S1–S6) with S1–S4 forming a voltage sensing domain and S5–S6 forming the central pore. Many channels also contain an associated regulatory domain at the cytosolic site [136]. 

Binding of spider neurotoxins to voltage gated ion channels was predominantly studied for Na_V_ channels. A recent 3D structure of a toxin-receptor complex is shown in Figure 3B. Most toxins were shown to bind to the helices S3 and S4 of the voltage sensing domain, allosterically modulating pore gating [137,138]. This is in contrast to the action of venom acylpolyamines, such as argiotoxin-636 (from *Argiope lobata*), which inhibits calcium-permeable AMPA receptors by directly inserting into the pore [139]. Toxin-channel interaction may also involve domains in proximity to S3 and S4 (e.g., S1–S2 loop, and S5), as recently shown for the double ICK peptide Dc1a bound to a mammalian Na_V_ channel [124]. Binding is often based on interaction of certain residues of the hydrophobic patch and the surrounding positive charged amino acids with hydrophobic and anionic amino acids of the target channel (compare Figure 3B1) [128]. A similar toxin surface was also shown to be involved in binding of the ASIC channel inhibitor PcTx 1 (pi-theraphotoxin-Pc1a, P60514) [140,141]. In contrast to voltage gated channel modulators, whose binding sites on channels are partially located within the membrane, PcTx 1, binds 45 Å above the lipid bilayer [140]. Intriguingly, PcTx 1 was anyhow shown to superficially interact with membranes. It has been proposed, that this interaction may be advantageous for finding its target channel due to reduction of the dimensions of diffusion [141].

#### 2.4.5. Modular Toxins—Combination of Toxin Structures

Most spider venom toxins can be either classified as “classic” AMP or “classic” neurotoxin-like peptide. Some toxins, however comprise a multi-domain construction with each domain resembling the structure of a “classic” spider toxin [142]. Multiple combinations of domains are thereby possible: neurotoxin-AMP, AMP-neurotoxin, AMP-AMP, and neurotoxin-neurotoxin.

**Figure 4:**
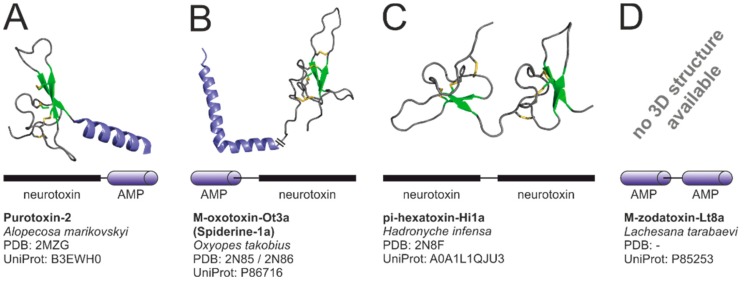
3D structures and schematic structures of modular toxins (multi-domain toxins). Antimicrobial domains are highlighted in blue, disulphide bridges in yellow. (**A**) Neurotoxin-AMP. (**B**) AMP-neurotoxin. The structure of spiderine-1a is assembled from two experimental 3D structures of the N- and C-terminal toxin part, respectively. (**C**) Neurotoxin-neurotoxin. (**D**) AMP-AMP.

Toxins exhibiting neurotoxic and cytolytic domains and corresponding activities exist in two forms. The cationic anti-microbial domain may locate C-terminal (e.g., CsTx-1, *Cupiennius salei* [20]; purotoxin-2, *Alopecosa marikovskyi* [143]) or N-terminal (spiderines, *Oxyopes takobius* [142,144]) of the neurotoxic domain (Figure 4A,B). Multiple assets from the two-domain construction of these toxins have been suggested. Oparin et al. [143] and Kuhn-Nentwig et al. [145] hypothesized that the α-helical part of these two-domain toxins binds to the cell membrane, thus increasing the efficiency of binding of the neurotoxic part to a receptor. Besides such a broader mode of action also an aggravation of the development of resistance against these two-domain toxins was suggested by Kuhn-Nentwig et al. [20]. The combination of cytolytic and neurotoxic activities of toxins is not restricted to spiders, but was also reported for scorpions [146].

Toxins with two consecutive ICK domains are described from four venom peptides of *Hadronyche infensa* (Hi1a–Hi1d) (Figure 4C). Each domain is highly similar to a known ASIC1a inhibitor. This is why the tandem structure of these toxins is thought to be a result of a duplication event of a gene encoding a single ICK gene. Hi1a was found to delay the activation of ASIC1a, a channel involved in stroke-induced neuronal damage, making it a promising candidate for development of neuroprotective stroke medication [147]. Toxins with similar double-ICK construction were also reported for the theraphosid *Cyriopagopus schmidti* (tau-theraphotoxin-Hs1a) [117] and *Cheiracanthium punctorium* (CpTx1–4) [148,149]. CpTx1–4 comprise at least 1/3 of all venom proteins of *Cheiracanthium punctorium.* It is likely, that CpTx1–4 additionally contain an α-helical motif C-terminally of the ICKs, making them similar to two joined neurotoxins with an additional cytolytic domain. 

Besides toxins with two neurotoxin-like domains, there are also toxins with two joined cytolytic domains (Figure 4D): The zodariid *Lachesana tarabaevi* expresses cyto-insectotoxins comprising two separated domains with a high propensity to form amphiphilic α-helices in proximity to membranes. These toxins show increased insecticidal activity compared to “simple” cytolytic peptides of the same spider [150].

### 2.5. Enzymes and Proteins

For a long time, enzymes and proteins were assigned a minor role in many spider venoms. Most publications constrained to enzymes described to target the extracellular matrix or the membrane of cells, facilitating the movement of toxins in the prey as so-called spreading factors. These enzymes include hyaluronidases, collagenases, and phospholipases. Hyaluronidases cleave the 1-4-linkages between N-acetylglucosamine and glucoronate in hyaluronan, a key component of the extracellular matrix. Another key component of the animal extracellular matrix, collagen, is cleaved by matrix-metalloproteases named collagenases. Phospholipases directly target the cells’ lipid bilayer by hydrolysis of phospholipids [14]. While many spider venoms contain phospholipase A [14], sicariid spiders contain extraordinarily high venom concentrations of phospholipase D (also referred to as sphingomyelinase D) [151]. This enzyme is responsible for necrotic effects in humans and efficiently immobilizes insects, also due to an additional neurotoxic effect [152]. 

In recent past, an increasing number of additional spider venom enzymes have been found and suggest a separation of spider venom proteins into three functional classes. (1) Enzymes acting as spreading factor (see above), (2) proteins with a function in the venom gland of the spider including the maturing of toxins (see next chapter), and (3) proteins directly targeting important systems of the prey organism. Functions of venom proteins, however, are in most cases not experimentally studied, but only hypothesized based on similarity to known enzymes.

Proteins with a potential function in the venom gland include protein disulphide isomerases [107], a peptide isomerase [153], carboxypeptidases [107], and the recently identified 28 kDa serine proteases responsible for propeptide cleavage of immature toxin precursors (see next chapter). Other proteins, such a leucine-rich repeat domain-containing protein or a tachylectin 5A-like protein, showing similarity to immune active proteins of spider-related animals, were hypothesized to possibly be involved in protection of the venom gland against microbial infections [107].

Proteins probably directly targeting important systems in the prey may be large neurotoxic proteins (see below), an acetylcholinesterase responsible for degradation of the neurotransmitter acetylcholine at synapses [21], or an α-amylase, hypothesized to cause hyperglycemia in the prey due to fast release of high amounts of glucose from the prey’s glycogen storages [107].

Proteins with a high similarity to angiotensin-converting enzyme, an exo-metallopeptidase processing peptide hormones involved in blood pressure regulation of vertebrates, were identified in various venoms of spiders [107,154,155] and scorpions [156]. These enzymes could be involved in venom peptide processing or target peptide hormones in the prey [107]. Another group of frequently described spider venom proteins are cysteine-rich secretory proteins (CRISPs) homologues [21,22,107]. CRISPs belong to the CAP (cysteine-rich secretory proteins, antigen 5, and pathogenesis-related 1 proteins) protein superfamily and are widely distributed among animal venoms, e.g., scorpions and snakes [157], but are also found in mammalian epididymis and the immune system [158]. Many different functions are described for CRISPs, including involvement in sperm-egg fusion [159] and immune defense [160] for mammalian CRISPs, or ion channel blocking effects for snake venom CRISPs [161,162,163]. In addition, a CAP superfamily saliva protein of blood-sucking insects was recently described to inhibit platelet aggregation and act as antioxidant enzyme by scavenging of superoxide [164]. The function of CRISPs in spider venom, however, remains elusive.

The majority of the here described venom proteins act as enzymes. A notable exception are latrotoxins, a group of neurotoxic proteins with masses between approx. 110 and 130 kDa initially reported from widow spiders (*Latrodectus* species) [165]. Similar toxins were later also reported from the venom of *Steatoda* and from genomic data of *Parasteatoda* (both Theridiidae), but the presence in the genome does not necessarily imply expression in the venom gland [166,167,168]. Latrotoxins exert different specificities, for insects, vertebrates, or crustaceans, and are accordingly named latrotoxins (against vertebrates), latroinsectotoxins, or latrocrustotoxins. The best studied latrotoxin is alpha-latrotoxin, which can cause fatalities in vertebrates. The main toxic action of alpha-latrotoxin relies on the formation of homotetrameric complexes that insert into presynaptic neuronal membranes, forming cation permeable pores [169,170]. Pore formation leads to massive influx of Ca^2+^, stimulating the release of all known types of neurotransmitters from presynaptic cells ultimately leading to the blockade of signal transmission inducing clinical symptoms such as muscular paralysis [81]. Binding of alpha-latrotoxin to membranes is thought to be mediated through surface proteins. Three alpha-latrotoxin receptors could so far be identified: Neurexin, latrophilin, and tyrosine phosphatase σ. Alpha-latrotoxin may also induce neurotransmitter release through a pathway, which is independent from influx of Ca^2+^ into the presynaptic cell (for a review see Yan and Wang [171]). Latrotoxins comprise a common structure featuring an N-terminal part with conserved regions, a central part containing multiple ankyrin repeats, and a C-terminal propeptide [165]. Ankyrin repeats mediate protein-protein interactions and are one of the most frequently observed amino acid motifs [172]. Posttranslational removal of the C-terminal propeptide seems to be required to exceed a toxic action as propeptide containing precursors of δ-latroinsectotoxin from *Latrodectus tredecimguttatus* have been shown to be inactive [173].

### 2.6. Spider Venom Peptide Precursors and Their Maturing

**Figure 5:**
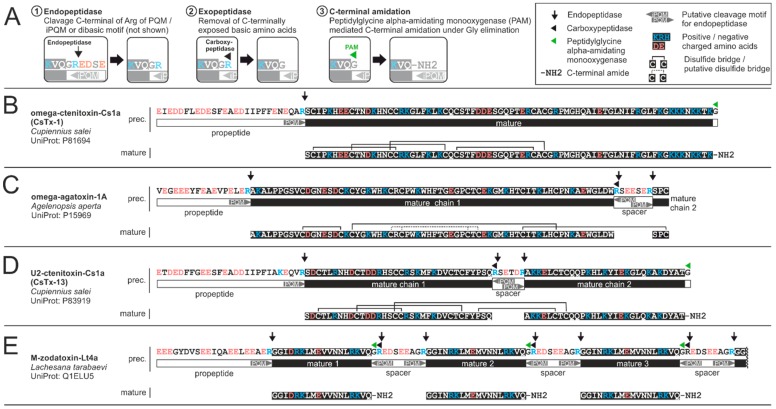
Maturing of venom peptides. (**A**) Stepwise schematic representation of the general maturing processes on the example of CsTx-13 (see panel D). (1) The Processing Quadruplet Motif (PQM) protease cleaves C-terminal of the Arg residue of the PQM or inverted Processing Quadruplet Motif (iPQM). (2) A so far uncharacterized carboxypeptidase subsequently removes the C-terminally exposed Arg if present, and (3) if a C-terminally exposed Gly is present, this is eliminated under formation of a C-terminal amide. (**B**–**E**) Schematic representation of chosen precursor sequences (top) and the corresponding mature sequences of neurotoxins. Maturing processes are indicated by arrows and triangles (see legend in the top right corner). (**B**) Precursor of a monomeric neurotoxin. (**C**,**D**) Precursors of heterodimeric neurotoxins. (**E**) N-terminal segment of a complex AMP precursor. Amino acids with positive side-chain charges at pH 7 are shown in blue, such with negative charges at pH 7 in red. All here shown precursors also comprise signal peptides which are not shown for reasons of space.

Neurotoxic and cytolytic spider venom peptides are expressed as precursors undergoing proteolytic cleavage to yield the mature peptide. Precursors of neurotoxic peptides typically comprise three consecutive parts. (1) A signal peptide, which is removed during translocation through the endoplasmic reticulum, (2) a propeptide, and (3) the mature toxin. Most propeptides feature lengths between approx. 6 and 30 amino acids and commonly contain a high number of negatively charged amino acids often compensating for the overall positive charge of the mature peptide (Figure 5) [107]. It has been proposed, that this charge compensation is beneficial for the storage of the immature toxin, as electrostatic repulsion between the precursors is reduced [107]. Propeptides may also assure correct peptide folding, sorting, and maturation [81] and prosequence elements of AMPs may neutralize their lytic activity before cleavage [174].

In 2005, Kozlov et al. [17] recognized that the four C-terminal amino acids of the propeptide form a conserved motif. This motif comprises an arginine residue at position −1 and at least one glutamic acid residue at positions −2 to −4 before the start of the mature peptide. The motif, named Processing Quadruplet Motif (PQM), is thought to act as protease recognition site for the propeptide cleaving protease. Later, a minority of neurotoxic peptide precursors were found to feature a dibasic cleavage motif (e.g., KR, RR) instead of a PQM, or not to comprise any propeptide [145,175].

Antimicrobial peptides show a precursor organization comparable to neurotoxic peptides comprising a signal peptide followed by an acid propeptide and the mature peptide. Besides these simple precursors, there are also binary or complex precursors, comprising multiple mature AMPs in a row, separated by short spacer sequences (Figure 5E) [176]. The spacer sequences thereby contain protease recognition motifs for posttranslational cleavage, allowing release of the individual AMPs from the long precursor. The recognition motifs are usually a PQM at the C-terminal end of the spacer and a so-called inverted Processing Quadruplet Motif (iPQM) at the N-terminal site of the spacer. An iPQM is basically a mirrored PQM comprising an Arg residue at position 1 and at least one Glu residue at positions 2 to 4. During removal of spacers from the complex precursors, the PQM is cleaved C-terminally of its Arg-residue in analogy to the propeptide cleavage. iPQM cleavage, however is proposed to be a two-step processing process, comprising first cleavage C-terminally of the Arg residue of the iPQM by an endopeptidase, and second, removal of the remaining Arg-residue by a carboxypeptidase (Figure 5A) [177]. The endopeptidase involved in PQM and iPQM cleavage is thereby likely to be the same [178].

Expression of AMP-precursors featuring a propeptide has been proposed to be a way for preventing potential harmful cytolytic actions during the biosynthesis of a toxin. It is believed that the negatively charged propeptides or spacers thereby interact with the positively charged clusters of the mature toxin neutralizing its effect before the toxin is activated by the processing into its mature form [74,174]. At which state of venom peptide production the protease-mediated maturing occurs (e.g., before or after storage in secretory vesicles) remains uninvestigated. However, the existence of heterodimeric toxins with disulphide linked chains as described below implies a precursor cleavage after the disulphide bridge formation.

#### 2.6.1. Comparison of Protease Cleavage Motifs to Motifs of Other Organisms

Distinguishing between cleavage motifs can be challenging, as certain sequences may be following the rule of several described motifs (e.g., EVKR may be classified as dibasic motif and as PQM). Dibasic cleavage motifs and similar motifs comprising a basic residue at the cleavage site and another one 0 to 6 amino acids before, are widespread among neuropeptides, hormones and growth factors of different organisms. This motifs are cleaved by subtilisin-like proprotein convertases (calcium dependent serine proteases) and the resulting C-terminally exposed basic amino acids are subsequently removed by metallo-carboxypeptidases [177,179,180,181]. Dibasic motifs or motifs with an extended distance between the two basic residues have also been found in precursors of peptides from sea anemones, scorpions, snakes and amphibians. Few of these motifs also feature Glu-residues in the range of +4 or −4 amino acids from the cleavage site, making it possible to be additionally classified as PQMs and iPQMs [177]. Unambiguous PQMs are rarely present in precursor sequences (e.g., P86399). Thus spider venom peptide maturing seem to rather represent an exception compared to the maturing of venom peptides of other animals [81]. 

Toxin precursors of other animals may also feature a construction different from the classic spider venom peptide precursor. Scorpion toxin precursors, for example, may be free of propeptides, contain propeptides N-terminally of the mature peptide [86,182], or contain propeptides C-terminally of their mature toxin [183,184]. We are not aware of C-terminal propeptides in spider venom peptide precursors. Gomesin, however, an AMP isolated from spider hemocytes, contains a C-terminal propeptide separated by a dibasic motif from the mature chain [185].

Production of toxins through complex toxin precursors, giving rise to multiple mature peptides, is not a singularity of spiders, but has also been described for other organisms including the toad *Bombina maxima* [186], the sea anemone *Urticina grebelnyi* [187], and some centipedes [188]. Complex structures of AMPs of the immune system are, among others, known from the honey bee [189], and from plants [190].

#### 2.6.2. Cleavage for Heterodimerization of Spider Toxins

Today, we know only a few heterodimeric neurotoxin-like peptides from spider venom. CsTx-13 (U2-ctenitoxin-Cs1a, P83919) from the spider *Cupiennius salei* is an ICK-motif peptide with two inter-chain disulphide bridges (Figure 5D) acting synergistically with its main monomeric neurotoxin CsTx-1 (omega-ctenitoxin-Cs1a, P81694) and its main cytolytic peptide cupiennin 1a (M-ctenitoxin-Cs1a, P83619) [59]. In CsTx-13, the two chains are of similar length. The only other well characterized heterodimeric spider venom toxin, omega-agatoxin-1A (P15969) of the agelenid *Agelenopsis aperta*, in contrary, possesses a very short chain consisting of only three amino acids connected with a disulphide bridge to a longer chain (Figure 5C) [191]. A homologue of omega-agatoxin-1A has also been found in the venom gland transcriptome of *Cupiennius salei* (CsTx-40, MH754614) [107,178]. All these peptides undergo heterodimerization by proteolytic cleavage of a monomeric precursor. The precursor comprises both mature chains separated by short spacers (RSETDR, RSEESER, and RNEEAER, respectively). The evident similarity of the precursor structure to the precursors of binary and complex precursors of AMPs suggests an analogue maturing mechanism by cleavage of the PQM and iPQM followed by removal of the residual Arg at the C-terminal end of the chain 1 [177,178].

Besides protease mediated cleavage of the peptide precursors, spider venom toxins may undergo additional posttranslational modification. Commonly observed modifications include removal of C-terminal Arg-residues by a so far uncharacterized carboxypeptidase, and peptidylglycine alpha-amidating monooxygenase mediated C-terminal amidation of peptides (Figure 5) [107]. In addition, pyroglutamate formation at N-terminal glutamine, formation of O-palmitoyl threonine, and isomerization of an amino acid have been described [176,192,193].

### 2.7. Concluding Remarks on Venom Components and Their Toxic Action

Here, we reviewed spider venom components in groups (i.e., small molecular mass compounds, AMPs, cysteine-rich peptides, and proteins). Some venom components known today cannot be assigned to any of the above groups. Among them are short peptides with similarity to peptide-hormones of the kinin family, such as tachykinin-like short cationic peptides from the ctenid *Phoneutria nigriventer*, that provoke smooth muscle contractions in guinea pig ileum [194], or bradykinin-potentiating peptides from *Lycosa erythrognatha* (mentioned as *Scaptocosa raptoria*) [195,196] and *Latrodectus tredecimguttatus* [197]. Some of these peptides have been shown to relax rat duodenum, increase capillary permeability, and to inhibit angiotensin converting enzyme [196].

Despite the here presented description of the toxic action of single venom components or groups of venom components, the importance of synergistic effects for the overall toxicity of venom should not be underestimated. Examples for reported synergistic actions are enhanced toxicity of neurotoxins in presence of potassium ions [58] or synergistic actions between the heterodimeric neurotoxin CsTx-13 (U2-ctenitoxin-Cs1a, P83919, *Cupiennius salei*) and the main monomeric toxin of the same spider (CsTx-1, omega-ctenitoxin-Cs1a, P81694) [59]. In addition, neurotoxins with different functions may interplay to ensure efficient effects in the prey. For example, it can be assumed, that the δ-toxins, and the κ-toxin co-occurring in the venom of the theraphosid *Chilobrachys guangxiensis* preserve action potentials (delayed inactivation of Na_V_ channels through δ-toxins) and simultaneously prevent their termination (blocking activation of K_V_ channels by κ-toxin) leading to tetanic paralysis [198]. Synergistic actions in insecticidal activity have also been reported for AMPs and neurotoxins [52,73].

Additionally, it has been shown, that neurotoxins have a stronger affinity for ion channels, if the lipid membrane contains more anionic ceramide 1-phosphate, than zwitterionic sphingomyelin. Sphingomyelin is hydrolyzed to ceramide 1-phosphate by sphingomyelinase D, an enzyme found in the venom of sicariid spiders, what implies a synergistic effect between neurotoxins and sphingomyelinase D [199,200]. Synergistic actions are also assumed for glutamate and acylpolyamines. Acylpolyamines block ionotropic glutamate receptors by inserting into the activated channel. Venom glutamate activates these channels upon venom injection and thus allows efficient blockade [81]. 

The here mentioned synergistic actions between venom components are only the tip of the iceberg and the interplay between venom components has only been marginally studied today. This especially concerns venom proteins that have only recently gained in focus [107]. Furthermore, it can be assumed that venom components may also have different purposes, sites, and time-courses of action [198]. Some components are likely to induce pain and mainly have a defensive purpose (e.g., ATP, serotonin, histamine), others ensure reversible, but immediate paralysis, or traverse the blood-brain barrier to act directly on the CNS and induce irreversible flaccid paralysis 20 to 30 minutes after injection [198]. All in all, the envenomation process seems to rely on a complex interplay of different venom components, which cannot be fully understood by individual characterization of the components. In the words of the Greek philosopher Aristotle, one may state that venom is more than the sum of its parts.

## 3. Analysis of Venom Components

Spider venom research started mid of the 20th century [201]. First studies were mainly performed with venom as a whole and especially concerned the effect of spider venom on animals and humans including the development of antivenin [202]. Venom was obtained by electrical milking, similar to the method which is still widely used today [203]. The small quantities of gained venom were for a long time a limiting factor for analysis of single venom components. Only after the advent of modern chromatographic separation techniques (especially high-performance liquid chromatography, HPLC), scientists were equipped with the appropriate tools for efficient isolation of toxins from small quantities of venom [34]. In the following years, multiple single peptide compounds and SMMCs were described or isolated from venoms. Multidimensional liquid chromatography was the method of choice for isolation of peptides [50,204,205,206]. Peptide sequences were often analyzed by Edman degradation and SMMCs were investigated by methods including mass spectrometry [206,207] and thin layer chromatography [50]. 

More and more emphasis was put on the development of drugs and insecticides from venom components [208,209]. Venom components with activity against a desired target started to get identified by assay guided fractionation using screening assays relying on methods as automated-patch-clamp electrophysiology, or fluorescent-resonance energy transfer [210]. Upcoming DNA sequencing techniques and soft ionization techniques for mass spectrometry (i.e., matrix-assisted laser desorption/ionization (MALDI), and electrospray ionization (ESI)) accelerated venom research and offered insights into venoms, that were only available in very limited quantities. 

Researches started to investigate sequences of single transcripts [211,212]. This was followed by generations of whole venom gland cDNA libraries, first sequenced by Sanger sequencing [16,17,18,19], later by Roche 454 sequencing [20,21], and Illumina sequencing [22,24]. Transcript sequences started to get used for cloning, design of mutants and heterologous expression, or chemical toxin synthesis to gain new structural and functional information (for a recent review see [210]).

Also in the last decade, mass spectrometry gained in importance for protein analyses and was increasingly used to study venom peptides and proteins. While analysis were first mainly restricted to purified components, rapid advances in detection speed, sensitivity, and coupling of liquid chromatography to mass spectrometers enabled automated studies of complex mixtures of peptides and proteins. An increasing number of transcriptomic studies were also paired with proteomic studies offering complementary data. Today, integration of proteomic, genomic and transcriptomic approaches in venom studies are termed *venomics* [213].

Transcriptomics and mass-spectrometry based proteomic studies are common in spider venom research and are further detailed in the following paragraphs. Genomic studies of spiders are rare, with only four genomes published so far (as of 9 July 2019): *Stegodyphus mimosarum*, *Acanthoscurria geniculata* [214], *Nephila clavipes* [215], and *Parasteatoda tepidariorum* [167,216]. Additionally, three genomes are deposited on the NCBI genome repository without any linked publication: *Dysdera sylvatica*, *Latrodectus hesperus*, and *Loxosceles reclusa*. For spiders, genome sequencing and analysis has been reported to be very challenging due to the high amount of repetitive elements likely to be a result of gene and whole genome duplications [216,217]. These repetitive elements enormously complicate de-novo assembly of reads. In this respect, the inclusion of data from long-read sequencing methods (see below) has been proposed to overcome fragmented genome assemblies [215,217]. More high-quality genomic data could thereby dramatically increase our understanding of the evolution of spider venom components. 

### 3.1. Venom Gland Transcriptome Analysis

Analysis of the venom gland transcriptome offers insight into the composition of protein and peptide venom components and is the method of choice to study venom components if the availability of venom is poor. In the early phase of transcript analysis, cDNA sequences were sequenced in targeted approaches. The probably first transcriptomic analysis of spider venom toxins, performed by the group of the Russian researcher Eugene V. Grishin, investigated the coding sequence of alpha-latrotoxin by targeted sequencing of the respective clone from a cDNA library. The clone was identified by hybridization of a synthetic oligonucleotide based on the sequence of a tryptic peptide of alpha-latrotoxin [212]. Later, multiple studies used rapid amplification of cDNA ends (RACE) [218,219,220], requiring a specific oligonucleotide for amplification of the cDNA of interest (3’ RACE) or for reverse transcription of the transcript of interest (5’ RACE) [221]. These labor-intensive targeted sequencing approaches were soon replaced by random screening of cDNA libraries, allowing parallel analysis of numerous venom peptide and protein coding transcripts. 

Sequencing techniques are under fast development and a variety of different techniques have been used for analysis of spider venom gland transcriptomes. As of 2 September 2019, 44 spider venom gland samples are listed in the biological source material database BioSample (search: “Araneae” [Organism] AND (venom [All Fields] AND gland [All Fields]; entries manually reviewed). Thereof, 12 samples have linked entries in the expressed sequencing tag database (dbEST) arising from Sanger sequencing, and 32 in the short read archive (SRA), a repository for next generation sequencing data. Many of the here deposited data, however, are not linked to and analyzed in original research papers. Table 2 gives an overview on published transcriptomic data from spider venom glands. 

First venom gland transcriptomes were analyzed by Sanger sequencing [16,17]. mRNA was isolated, reverse transcribed, cloned into plasmids, amplified in bacteria, and finally the inserts of plasmids were sequenced by Sanger sequencing. This method generates sequences of up to 900 nucleotides in length, referred to as expressed sequencing tags (ESTs) [243]. Depending on the length of a transcript, ESTs may not span an entire transcript, but only represent portions of it. An attempt to decrease the data complexity for downstream analysis is to assemble ESTs belonging to the same mRNA to so called contigs. This computational process, however, is not trivial and may produce artefact contigs that do not represent real mRNAs. The availability of reference sequences, such as a reference genome, can therefore significantly improve transcriptomic analysis as ESTs do not need to be de-novo assembled, but can simply be mapped to reference sequences. High-quality spider genomes, however, are extremely rare and sequencing reads are usually de-novo assembled. De-novo assembly quality is significantly influenced by the length of the assembled sequencing reads with longer reads, resulting in better assemblies [210]. Development of next generation sequencing techniques, such as 454 sequencing or Illumina sequencing drastically increased the number of generated sequencing reads, allowing to generate more comprehensive transcriptomic data and to better estimate transcript abundance from the number of respective reads. However, these advances came at the cost of worse accuracy and shorter sequencing reads (e.g., depending on the protocol, mostly 150 base pairs (bp) for Illumina or up to 700 bp for 454 sequencing [243]) complicating de-novo assembly, especially for Illumina data. While Sanger sequencing of venom gland transcriptomes often resulted in the generation of approx. 100 contigs and somewhat more ESTs, that could not be assembled (referred to as singletons), reads from next generation techniques are often assembled to several thousands of contigs (Table 2), requiring bioinformatic tools to identify sequences of toxin-like peptides from the database of contigs.

#### 3.1.1. Identification of Toxin-Like Transcripts

Different strategies have been used to identify toxin-like peptides from transcriptomic data (Table 2). The most widely used approach is Basic Local Alignment Search Tool (BLAST) of contigs against specialized databases containing known toxins, such as ArachnoServer [46,47], the animal toxin database of UniProt (ToxProt), or more comprehensive databases, such as UniProtKB. Many studies also incorporate protein domain prediction and gene ontology annotation of contigs. Hits are typically searched for toxin related terms and are manually reviewed. While many studies focus on neurotoxin-like peptides, structural features of the latter are also commonly used for toxin identification. These include cysteine count, cysteine pattern search, and presence of signal and/or propeptide cleavage sites. Prediction of possible propeptide cleavage sites was significantly improved with the publication of spiderP and its successor spiderProHMM, both automated propeptide prediction software for spider toxins. Many of the above-mentioned approaches assume a certain degree of similarity of newly identified toxin-like peptides with known toxins. This makes it challenging to identify entirely new toxin families. In addition, as toxins are derived from body peptides, simple homology search approaches (e.g., BLAST) involve the danger of identifying body peptides and wrongly annotating them as toxins (i.e., wrong positive) [244]. The number of false positive identifications may be diminished by integration of proteomic data, phylogenetic analysis of putative toxin families, and/or the use of comparative transcriptomics of non-venom producing tissues and venom gland [244,245]. Accordingly, Haney et al. [22] used a multi-tissue sequencing approach to investigate the venom of the spider *Latrodectus hesperus* and sequenced mRNA isolated from venom gland, silk gland, and the prosoma without the venom gland, allowing to identify transcripts with venom gland biased expression.

Comparison of transcriptome derived toxin-like sequences with transcriptomic data of haemocytes was recently used to reinforce venom gland specific expression of single toxins of *Cupiennius salei* Kuhn-Nentwig et al. [107], and Huang et al. [224] compared tissue specific expression of toxin-like peptides and proteins identified in the venom gland transcriptome of the lycosid *Pardosa pseudoannulata*. Putative cysteine-rich venom peptides were found to be uniquely or predominantly expressed in the venom glands while some putative venom protein coding transcripts showed surprisingly high expression levels in brain tissue or fat body. This demonstrates the complexity to accurately predict venom proteins from transcriptomic data.

The identification of toxin-like sequences may be influenced by the protocol of data analysis. Different protocols for data acquisition, however, may also influence toxin recovery and quantification. For example, transcript levels may be influenced by the duration between the last venom gland depletion by milking or feeding, and the dissection of venom glands. Many studies report a timeframe between 48 h and one week from last milking to dissection of the venom glands (Table 2). Venom gland dissection is even performed at different time intervals after milking in some studies to diminish the potential effects of a time-dependent expression of certain venom components. Experimental data on time-dependent expression of venom peptides and proteins, however, is lacking. 

Toxin discovery may also substantially depend on the used sequencing method. Comparing 454 with Illumina sequencing, the greater read depth of Illumina has been shown to improve toxin discovery. In a recent studies of the venom of the remipede crustacean *Xibalbanus tulumensis,* 23 of 32 toxin families were only identified from Illumina data [246], and for *Conus tribelei*, 10 of 30 conopeptide superfamilies were only identified from Illumina data [247]. Interestingly however, for the spider *Phoneutria nigriventer*, Diniz et al. [154] identified more toxin-like peptides with traditional Sanger sequencing than with Illumina sequencing.

#### 3.1.2. Recent Developments in Sequencing Techniques

DNA sequencing techniques are currently under constant development. Meanwhile techniques for single molecule sequencing, such as Oxford Nanopore Technologies (Nanopore), and Pacific Bioscience (PacBio), termed third generation sequencing, are increasingly available. To our knowledge, these techniques have so far not yet been applied for spider venom gland studies. With read lengths between 10,000 and 100,000 bases, third generation sequencing techniques produce much longer reads than next generation sequencing [248]. These read lengths enable full-length mRNA sequencing overcoming problems in de-novo assembly often encountered in next generation sequencing protocols. Full-length mRNA sequencing also provides a significant advantage for quantification of transcripts of closely related isoforms [249]. This can be challenging with short read methods as a read may not be unambiguously assigned to a single isoform. 

Third generation sequencing methods, however, have two major drawbacks. (1) High error rates of up to 15% (compared to approx. 0.1% for Illumina), and (2) low throughput compared to Illumina sequencing [249]. These drawbacks can be diminished if data from third generation sequencing is jointly assembled with data from a next generation sequencing technique [250,251]. This hybrid assembly approach, has been shown to facilitate de-novo genome assembly, and may also be applicable to study genomic data of spiders, which has up to now been a challenging task due to many repetitive elements, difficult to assemble from short reads. Third generation sequencing techniques may also drastically facilitate analysis of highly repetitive transcripts, such as the ones coding for complex cytolytic precursors (see above). The attractiveness of using PacBio alone for de-novo genome or transcriptome sequencing was very recently enhanced with the publication of a circular consensus sequencing protocol allowing reduction of error rates to <1% for single-molecule reads of up to 15 kb length [252,253].

In spider venomic studies, third generation techniques have not established yet and many recently published data were still acquired with next generation techniques or conventional Sanger sequencing. While 454 sequencing was meanwhile discontinued by Roche, Illumina sequencing stays widely used and gets further developed with the only recently released NovaSeq 6000 sequencing system. 

#### 3.1.3. Cross Contamination among Multiplexed Samples in Illumina Sequencing

Illumina is a sequencing by synthesis method relying on detection of fluorescent tagged nucleotides. Synthesis is performed from templates immobilized on the surface of a so-called flow cell. One flow cell thereby features several lanes (e.g., eight for Illumina HiSeq4000), in which the sequencing reaction is performed physically separated. One lane generates up to 280 million reads of 125 bp if sequenced with the meanwhile discontinued Illumina HiSeq2500. The number of reads per lane is even higher in newer Illumina sequencing solutions, such as Illumina HiSeq4000, and Nova Seq 6000, that use a special flow cell surface (patterned flow cells). 

This high number of reads is an overkill for many analyses, however, economical use of sequencing capacities is possible using multiplexing, the sequencing of multiple different samples in one sequencing lane. For sample multiplexing, adapters with sample-specific indexes (barcodes) are attached to each sequencing fragment during the preparation of the sequencing library. Subsequently, multiple libraries can be pooled and sequenced in the same sequencing lane. The unique indexes are sequenced in a separate sequencing run and are used to reassign a sequence read to its respective sample in a process called demultiplexing. Despite the wide use of multiplexing, recent studies unveiled massive cross contamination between multiplexed samples caused by misassignment of individual reads to the wrong sample. This “index hopping” or “index swapping” was early described by Kircher et al. [254] who proposed the use of double barcoding (e.g., the use of two separate indexes per sample, introduced in the 3’ and 5’ adapter) to diminish the problem.

It took some years until Illumina itself and further scientists published studies concerning index misassignment. Today, Illumina states that the main underlying process is contamination from free adapters that were not completely removed during library preparation [255]. These adapters can lead to extension of library fragments with the wrong index adapter, an effect which is bigger if cluster generation is achieved via ExAmp chemistry used for patterned flow cells, as used for HiSeqX, HiSeq 4000/3000, and NovaSeq 6000. Illumina itself estimates the misassignment rates up to 2% and ≤1% for patterned and non-patterned flow cells, respectively [255]. Using patterned flow cells and single indexing, Costello et al. [256] reported 0.2% to 6% (average 0.89%) cross contamination by index misassignment, that can however be significantly reduced using non-redundant double-indexing and filtering of unexpected adapter combinations during demultiplexing. Even higher contaminations of 5% to 7% were reported by Sinha et al. [257]. Studies showed, that non-patterned flow cells performed way better with contaminations of 0.09% to 0.6% in MiSeq [256] or even complete absence of misassignment [257]. Interestingly, Griffiths et al. [258] reported up to 2.5% misindexing, however found free DNA barcode concentrations not to affect the rate of cross contamination. Owens et al. [259] could not find index swapping in data neither from HiSeq 2500 nor from HiSeq X. In summary, studies show different cross contamination rates of 0–10% but commonly propose to diminish cross contamination by use of non-redundant double barcoding and/or completely abandoning multiplexing [257,259].

The reported rates of cross contamination may be a big issue in experiments concluding results from very low expressed genes, such as some recent experiments in cancer genomics [256]. Non-realized cross contamination between multiplexed samples may also have a significant impact on the outcome in venomic studies. For example, multiplexing of venom gland transcriptome libraries of different organisms may lead to the wrong conclusion that these organisms share common venom peptides or proteins. Expression level estimates will surprisingly show that the related transcripts are low expressed in all but one organism. While the experienced scientist may recognize that the results as biased, especially if the phylogenetic distance between the multiplexed organisms is high, cross contamination in multiple tissue profiling experiments may be a bigger issue. Venom gland specific transcripts may be found at lower expression levels in other tissues as well. 

Interestingly, a similar case has been recently described in a study comparing the transcriptomes of venom glands, silk glands, ovaries, and the prosoma without the venom glands of *Parasteatoda tepidariorum* ([23] STable6). The authors reported that “in contrast with the selective expression in the venom gland as posited in the traditional model of venom evolution …, many of the individual transcripts that produce venom proteins in this study have some level of expression in other tissues (silk, ovary, or cephalothorax).” [23]. The exact sequencing conditions including multiplexing level and samples multiplexed are not disclosed. Upon request, the corresponding author stated that sequencing was done in a single lane of an Illumina HiSeq4000 instrument. Some libraries were re-sequenced in a different sequencing run under non-disclosed multiplexing conditions (personal communication, Haney 2019). In the light of the latest findings concerning cross-contamination in Illumina sequencing, it cannot be excluded that some these transcripts may rather be a result of cross-contamination than they are truly expressed in other tissues.

### 3.2. High Throughput Mass Spectrometric Analysis of the Venom Proteoms

For a long time, the method of choice for sequence analysis of venom peptides and proteins was purification of analytes and subsequent Edman degradation. This labor- and cost-intensive approach lost in importance with upcoming high-throughput mass spectrometric techniques [34]. Today, two main workflows are used in mass-spectrometry based comprehensive analyses of proteomes: (1) Tandem mass spectrometric analysis of fragments resulting from enzymatic or chemical digestion of proteins and peptides (bottom-up proteomics), and (2) direct analysis of intact proteins and peptides by tandem mass spectrometry (top-down proteomics).

#### 3.2.1. Bottom-Up Proteomics

Most proteomic studies of venoms use bottom-up approaches. Some studies involve extensive decomplexation of samples down to single peptides/proteins or groups of peptides/proteins prior to proteolytic digestion. A standard protocol developed for the analysis of snake venom starts with decomplexation of venom by fractionation through reverse phase HPLC (RP-HPLC). RP-HPLC fractions are subsequently separated by sodium dodecyl sulphate polyacrylamide gel electrophoresis (SDS-PAGE), single protein bands are excised from the gel, and proteins are in-gel digested with a protease (mostly trypsin). The generated protein fragments are analyzed by tandem mass spectrometry to gain sequence information. Relative abundances of venom peptides and proteins can be estimated from the peak integral of PR-HPLC peaks and/or the staining intensity of bands in SDS-PAGE. Another approach for offline-separation and relative quantification of venom components is 2D electrophoresis (isoelectric focusing and SDS-PAGE) of venom. This 2D gel-based approach, however, bears some constraints in quantification of very low or very highly expressed peptides and proteins due to the limited dynamic range of protein concentration that can be resolved by electrophoresis [260].

Extensive decomplexation of samples before proteolytic digest allows to gain further information of single peptides/proteins or groups of peptides/proteins. For example, gels may be blotted to a membrane for subsequent immunoblotting analysis using antivenoms [261,262]. However, decomplexation of venom components and handling of dozens of single samples for downstream mass spectrometric analysis is cost- and labor-intensive. Direct mass-spectrometric analysis of peptide and protein fragments resulting from proteolytic digestion of crude venom, or venom fractionated in only few fractions is significantly faster and easier to perform. In this approach, also termed “shotgun” proteomics, fragments resulting from proteolytic digestion of peptides and proteins are typically separated by capillary RP-HPLC and sequenced by on-line tandem mass spectrometry. Multidimensional separation of peptide and protein fragments (e.g., by ion-exchange chromatography and RP-HPLC) can thereby increase peptide resolution. Direct shotgun proteomics is highly sensitive and also reveals information on small peptides (<2–3 kDa), which may be lost in gel-based approaches. The major drawback, however, is the lack of easy possibilities for quantification of venom components. Isotope based methods for absolute quantification require sample spiking with one specific isotope labelled standard per quantified proteoform, which is not applicable [263]. Label-free quantification techniques based on fragment ion peak intensities may be used for the relative comparison of identical components among different samples (comparative proteomics), but are not suitable for quantification of different components in one sample [260]. Thus, shotgun-proteomics may be suitable to provide qualitative, but not quantitative information on venom components [230].

In all of the above-mentioned methods, proteolytically digested proteins and peptides are fragmented in a mass spectrometer (usually by collision induced decay, CID) to gain sequence information from fragment spectra. The several thousands of resulting fragment spectra are in-silico matched against a database of possible proteins. In the best case, this database consists of protein sequences deduced from transcriptomic or genomic data of the analyzed species. Lack of this data often limits the identification of single proteins and mostly only allows to assign proteins to a known protein family based on similarity of short sequence stretches. Protein families are thereby either identified by direct search of fragment ion spectra against a more comprehensive database (e.g., containing all spider proteins) or, allowing also identification of less similar proteins, by de-novo sequencing and subsequent search of de-novo sequenced fragments against a comprehensive database. 

The majority of recently published proteomic studies on spider venom are not quantitative and use shotgun approaches and species-specific databases for matching of fragment ion spectra (for an overview on selected studies see Table 3). Sequence databases are mostly derived from translated sequences of a transcriptome analysis performed in the same study and proteomic results are used for qualitative validation of peptides inferred from transcriptomic data. For identification of proteins and protein families, few studies used non-species-specific databases or de-novo sequencing. De-novo sequencing was used in combination with database searches by Duan et al. [236] and Liao et al. [264]. In the analyzed studies, components of crude venom were either directly digested or pre-separated (mostly by SDS-PAGE) prior to digestion and mass spectrometric analysis. Multiple studies combined different protocols for maximizing data output. Gel-based and direct crude venom analysis were used by Santana et al. [265], and Duan et al. [236]. Other studies used enzymes with different specificities to improve the protein coverage [107,266].

Incomplete protein coverage and ambiguity in assigning sequenced fragments to closely related protein isoforms is a major difficulty in bottom-up proteomics, especially in shotgun approaches. This not only affects proteoform identification but also MS-based quantification [1,267,268]. Differentiation of isoforms is only possible if a unique peptide for each isoform can be unambiguously sequenced. Incomplete sequence information also significantly complicates the identification of heterodimeric toxins, e.g., CsTx-13 (U2-ctenitoxin-Cs1a, P83919) or omega-agatoxin-1A (P15969), or of C-terminally truncated toxins, e.g., CsTx-2a or CsTx-2b (omega-ctenitoxin-Cs1a, P81694)). These shortcomings may be eliminated by the use of top-down proteomics.

#### 3.2.2. Top-Down Proteomics

In top-down proteomics, intact peptides and proteins are directly analyzed by fragmentation in the mass spectrometer without prior digestion. The analysis of intact peptides and proteins, significantly simplifies the identification of individual proteoforms with single nucleotide polymorphisms (SNP), alternative splicing variants, and post translational modifications [267,271]. A prerequisite for top-down analyses is the availability of a mass spectrometer with fast acquisition and high resolving power for precursor and especially fragment ion spectra. Commonly used are Fourier-transform ion cyclotron resonance and Fourier-transform orbitrap mass spectrometers [267]. Analytes are usually pre-separated by online RP-HPLC. However, this cannot completely isolate all proteoforms. A higher degree of pre-separation (e.g., by multidimensional HPLC) can increase the resolution and isolation of single proteoforms resulting in more protein identifications [271]. Classic CID and HCD (higher-energy collisional dissociation) may be used as fragmentation method, however electron-transfer dissociation (ETD) or combined fragmentation approaches such as electron-transfer/higher-energy collision dissociation (EThcD) are more popular, since they provide better protein coverage [267]. 

Top-down proteomics yields extremely complex spectra. Data analysis is performed in-silico and includes isotopic peak picking and deconvolution followed by matching of fragment spectra against a user-provided database of intact proteins, and statistical validation of protein identifications [271]. Top-down proteomics is especially suitable for analysis of peptides and small proteins up to approx. 30 kDa in mass [267] and analysis of high mass proteins is difficult [271]. Today, most venomic studies still mainly relay on bottom-up proteomics. Top-down approaches are increasingly used to complement bottom-up data of spider [21,107] and snake [272,273,274,275,276] venoms. 

Recently, Calderon-Celis et al. [277,278] showed that snake venom proteins can be absolutely quantified using a top-down proteomic approach. In their study, sulphur containing venom proteins were in parallel (1) quantified via inductive coupled plasma (ICP) mass spectrometry using ^32^S/^34^S isotope dilution and (2) identified by ESI QTOF-MS [277]. ICP-MS is a highly sensitive method normally used in inorganic elementary analysis. Single atoms are ionized in a plasma and subsequently analysed in the mass spectrometer. The generated ion-signal is thereby proportional to the ion concentration in a wide dynamic range allowing absolute quantification of the ion concentration if a sample is spiked with an isotopically enriched standard (isotope dilution) [263]. ICP-MS based quantification of venom proteins may provide a promising tool for further venomic studies, but it also reveals a major challenge ahead because the full separation of proteoforms prior to mass spectrometry is a prerequisite for ICP-MS based quantification [277].

### 3.3. Annotation of Spider Venom Toxins

In the last decade, advances in omics techniques lead to a rapid increase in toxin sequences deposited in online repositories. The main repositories for manually annotated and reviewed spider toxin sequences are ArachnoServer [47] and ToxProt, the animal toxin annotation project of UniProt [279]. Sequences from high-throughput transcriptomics are often deposited in nucleotide sequence databases.

Many venomic studies today group newly identified toxins into families of similar toxins. Family annotation is thereby conducted among toxins of a species and/or toxins are compared with entries of inter-species repositories and annotated according to their similarity to known homologues. Detection of homologue sequences is in many studies achieved by pairwise sequence comparison tools, such as the Basic Local Alignment Search Tool (BLAST). An alternative approach is the use of profile Hidden Markov Models (HMMs) for annotation of toxins to an existing protein or peptide family [107].

Such profile HMMs are statistical models often calculated from multiple sequence alignments of protein domains, families, or structural motifs. For every position of a sequence, HMMs contain probability scores for all possible amino acids, insertions, and deletions to occur at this given position. Thus, in contrary to BLAST, search of sequences against profile HMMs employs position specific scoring matrices. Penalties for mutations in conserved protein regions are higher than for mutations in highly variable regions. This results in higher sensitivities, particularly for sequences with distant similarities [280]. Multiple sequence alignments and related HMMs are deposited in online repositories, such as Pfam [281] and sequences of interests can be scanned against predictive models of multiple databases using InterPro [282]. ToxProt, the animal toxin annotation project of UniProt, involves family classification according to Pfam profile HMMs. 

In 2017, Koua and Kuhn-Nentwig found these family annotations not to comprise all known toxin sequences anymore and reported a high hierarchical heterogeneity in family and subfamily organization [283]. They published new spider venom specific HMMs which we recently further refined during our analysis of 50 spider venom gland transcriptomes. These profile HMMs enable comprehensive peptide family classification and comparison of large datasets of toxins of different species, thereby allowing new evolutionary insights into spider venoms. The mentioned toxin family classification system has been proposed to complement the currently valid rational toxin nomenclature by King et al. [119] to easily infer structural similarities of toxins [283].

### 3.4. Concluding Remarks on the Analysis of Venom Components

In the last years the focus of venomic studies experienced a shift from single protein analysis to comprehensive assessment of the whole transcriptome and proteome, which is today possible with extreme sensitivity [263,284]. While spider venom proteomes have up to date mainly been qualitatively assessed, quantitative studies are more widespread amongst venomic analysis of other animals (e.g., snakes). Quantitative approaches have recently also been applied in top-down proteomics [277], the latest protocols in proteomic venom studies. Today, top-down approaches however seem still in their infancy and are more challenging to perform than bottom-up approaches. This is mainly due to fewer expertise, need for protocols for sample pre-separation and expensive high-resolution mass spectrometers, and less sophisticated data analysis tools [263,284]. Many advantages, such as gaining information about posttranslational modifications and qualitative discrimination between closely related proteoforms, however, predict the use of top-down approaches alongside with transcriptomic or genomic analysis a bright future in venomics [267,271]. In addition, integration of the newest single-molecule long-read sequencing techniques promise great improvements in de-novo read assembly, which is challenging with data generated with today’s widely used Illumina sequencing. Researchers working with Illumina data, should pay great attention to avoid multiplexing-based cross contamination, which could possibly affect the conclusions of their studies.

## Figures and Tables

**Table 1:** Activities and nomenclature [119] of main spider venom toxins.

Prefix	Target	Action	Example
ω (omega)	Ca_V_ channels	Inhibits Ca_V_ channels	omega-agatoxin-1a, P15969
κ (kappa)	K_V_ channels	Inhibits K_V_ channels	kappa-hexatoxin-Hv1c, P82228
β (beta)	Na_V_ channels	Shifts voltage dependence of Na_V_ channel activation	beta-hexatoxin-Mg1a, P83561
δ (delta)	Na_V_ channels	Delays inactivation of Na_V_ channels	delta-miturgitoxin-Cp1b, C0HKG8
µ (mu)	Na_V_ channels	Inhibits Na_V_ channels	mu-diguetoxin-Dc1a, P49126
M	membrane	Membranolytic activity	M-ctenitoxin-Cs1a, P83619
U	unknown	Unknown activity	U2-ctenitoxin-Cs1a, P83919

**Table 2:** Overview on selected transcriptomic studies of spider venom glands.

Spider Species	Sex/Number of Specimens/Time Since Last Milking	Sequencing Method	Total EST/Contigs	Isolation of Sequences Based on	Identified Toxins/Transcripts	Ref.
*Parasteatoda tepidariorum* */** (Theridiidae)	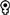 /adult/3 d since last meal, no milking	Illumina HiSeq 4000 (2 × 100 bp) 2 replicates	NA, aligned to genome assembly	differential expression analysis	1318 upregulated transcripts	[23]
*Phoneutria pertyi* (Ctenidae)	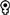 /10 ad./48 h	Sanger (1305 clones)	106 contigs, 189 singletons	Basic Local Alignment Search Tool (BLAST) (UniProtKB) non-hits manual	63 transcripts of cys-rich peptides	[222]
*Cupiennius salei* * (Trechaleidae)	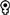 /20 ad./24, 48, 62 h; 8, 14 d	454 GS-FLX	34,107 contigs	BLAST (UniProtKB), signal peptide, HMM, cys-pattern	81 transcripts of cys-rich peptides, 56 mature peptides	[107]
*Selenocosmia jiafu* (Theraphosidae)	NA/6/4 d	Sanger (1299 clones)	752 ESTs, 61 contigs, 196 singletons	BLAST (UniProtKB, nrNCBI), signal peptide	257 transcripts, 99 mature peptides	[223]
*Tetragnatha versicolor* (Tetragnathidae)	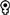 /10/2, 3 d 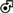 /2/2, 3 d analysed by sex	Illumina (2 × 50 bp) HiSeq 2500	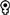 16,799 contigs, 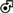 24,351 contigs	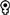 / 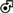 Reads remapped against combined assembly, TRINOTATE pipeline	NA, 9177 dimorphic, 1404 non-dimorphic	[35]
*Pardosa pseudoannulata* ** (Lycosidae)	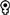 /15 ad./NA 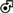 /15 ad./NA 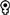 /45 ad./NA (tissue expression profiling)	Illumina HiSeq	75,980 contigs	BLAST (nrNCBI, ntNCBI, SwissProt), domain prediction, GO, cys pattern	48 potential peptide toxins	[224]
*Phoneutria nigriventer* * (Ctenidae)	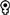 /20 ad./48 h 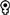 /10 ad./48 h	Illumina (2 × 151 bp) HiSeq 1500 Sanger (1476 electrograms)	49,992 contigs 1224 ESTs, 132 contigs, 162 singletons	BLAST (UniProtKB, TSA), domain prediction, GO, expression level BLAST (UniProtKB), ORF, signal peptide, propeptide	99 or 98 cys-rich peptide toxins	[154]
*Poecilotheria Formosa* * (Theraphosidae) *Viridasius fasciatus* * (Viridasiidae) *Latrodectus mactans* * (Theridiidae) *Heteropoda davidbowie* * (Sparassidae)	NA/9 ad./ 0, 2, 3, 4 d NA/10 ad./ 0, 2, 3, 4 d NA/39 ad./ 0, 2, 3, 4 d NA/9 ad./ 0, 3, 7 d	Ion Torrent	94,148 contigs 330,060 contigs 301,423 contigs 239,749 contigs	private HMMs based on ArachnoServer sequences, read count threshold	37 toxins 41 toxins 10 toxins29 toxins	[225]
*Cyriopagopus hainanus* (sub *Haplopelma hainana*) (Theraphosidae)	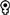 /1 ad./NA	Illumina (2 × 101 bp) HiSeq 2000	57,181 contigs	BLAST (Toxprot, UniProtKB), cys count ≥ 5, domain prediction	201 potential toxins	[226]
*Lycosa vittata* (Lycosidae)	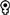 /6 ad./NA	Sanger (500 clones)	NA	NA	51 toxin-like peptides	[227]
*Cyriopagopus hainanus* (sub *Haplopelma hainana*) (Theraphosidae)	NA/NA/2 d	454 GS-FLX	65,432 contigs	BLAST (EST NCBI, nrNCBI), ORF, cys count ≥ 4, length ≥ 45 amino acids	1136 potential precursors	[228]
*Dolomedes sulfureus* (Pisauridae)	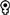 /10 ad./4 d	Sanger (500 clones)	267 ESTs, 25 contigs, 58 singletons	BLAST (nrNCBI, UniProtKB)	127 putative toxin precursors, 90 mature peptides	[229]
*Plectreurys tristis* * (Plectreuridae)	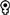 /5/3, 4 d	Sanger (1717 clones)	307 ESTs, 37 contigs, 105 singletons	BLAST (ArachnoServer, NCBI, private dbs)	19 putative peptide transcripts	[230]
*Cyriopagopus schmidti* (sub *Ornithoctonus huwena*) (Theraphosidae)	NA/3/2 d	454 GS FLX Titanium	4224 contigs	BLAST (UniProtKB, ToxRelDB, Repbase), cys pattern	626 toxin precursors, 90 mature peptides	[231]
*Latrodectus Hesperus* */** (Theridiidae)	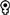 /7/NA	Illumina (2 × 100 bp)	85,193 contigs	BLAST (UniProtKB), ORF prediction, differential expression analysis	695 venom gland specific transcripts	[22] [232]
*Dolomedes fimbriatus* (Pisauridae)	NA/several/7 d	Sanger (5952 clones)	451 contigs	cys pattern search, signal peptide, propeptide	451 transcripts, 163 mature peptides	[233]
*Selenotypus plumipes* (Theraphosidae)	NA/2/NA	454 GS-FLX	136,469 six-frame translated sequences	ORF prediction, BLAST (ArachnoServer) signal peptide, propeptide, cys count	970 mature (likely to be an overestimate)	[234]
*Trittame lok i* * (Barychelidae)	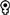 /9/4 d	454 GS FLX Titanium	4711 contigs	BLAST UniProtKB	46 full-length toxin precursors	[21]
*Dolomedes mizhoanus* (Pisauridae)	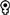 /1/4 d	Sanger	356 ESTs, 19 contigs, 26 singletons	BLAST (nrNCBI, UniProtKB) signal peptide, SpiderP	53 or 55 cys-knot toxin precursors, 48 mature peptides	[235]
*Latrodectus tredecimguttatus* (Theridiidae)	NA/3 ad./NA 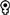 /15/4 d	Illumina (2 × 90 bp) HiSeq 2000 Sanger	34,334 contigs 1015 unique ESTs	ORF prediction, BLAST (UniProtKB), Cys-pattern, domain prediction (SMART/Pfam)	146 toxin-like proteins	[24]
*Araneus ventricosus* * (Araneidae)	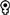 /20/3 d	Sanger	886 ESTs	≥4 cys, signal peptide	200 toxin-like precursors	[236]
*Grammostola rosea* (Theraphosidae)	NA/30/NA	Sanger (1500 clones)	869 ESTs	BLAST	48 peptides	[237]
*Lycosa singoriensis* (Lycosidae)	NA/20/4 d	Sanger	833 ESTs	BLAST (nrNCBI, UniProtKB)	223 toxin-like transcripts	[238]
*Cyriopagopus hainanus* * (sub *Ornithoctonus hainana*) (Theraphosidae)	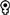 /20 ad./NA	Sanger (1049 clones)	NA	BLAST, pairing with proteomic data from N-term sequencing (Edman)	88 peptide toxins	[239]
*Loxosceles intermedia* (Sicariidae)	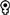 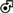 /350/5 d	Sanger (2400 clones)	1843 ESTs, 257 contigs, 281 singletons	BLAST(GenBank)	88 contigs, 80 singletons (toxin sequences)	[240]
*Pelinobius muticus* * (sub *Citharischius crawshayi*) (Theraphosidae)	NA/1/2 d	Sanger (282 clones)	236 ESTs, 14 contigs, 30 singletons	BLAST (GenBank, ArachnoServer)	11 toxin-like, 3 putative toxin transcripts	[241]
*Cyriopagopus schmidti* (sub *Ornithoctonus huwena*) (Theraphosidae)	NA/20/4 d	Sanger	468 ESTs, 24 contigs, 65 singletons	BLAST (nrNCBI, UniProtKB)	31 mature peptides	[242]
*Loxosceles laeta* (Sicariidae)	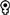 /100/5 d	Sanger	3008 ESTs, 326 contigs, 1031 singletons	BLAST, domain prediction (SMART/Pfam), signal peptide	93 clusters of known toxins, 117 clusters of possible toxins	[18]
*Agelena orientalis* (Agelenidae)	NA/NA/NA	Sanger (2166 clones)	37 contigs, 332 singletons	BLAST (GenBank, peptide sequence databases)	48 toxin-like structures	[17]
*Macrothele gigas* * (Dipluridae)	NA/2 glands/NA	Sanger (300 clones)	NA	NA	10 multi-cys peptides	[16]

* Studies include proteomic experiments; ** Studies include further tissues besides venom glands. The row *“Isolation of sequences based on”* describes the methods applied to retrieve toxin sequences from the contig database. Databases used for BLAST are given in brackets. Data not available from the cited reference is designated as NA (not available). The following abbreviations are used: TSA = transcriptome shotgun assembly protein database. GO = gene ontology annotation, ORF = open reading frame prediction, HMMs = Hidden Markov Models, ad. = adult, d = days, h = hour.

**Table 3:** Overview on selected high-throughput mass spectrometric studies of spider venom.

Spider Species	Top-Down	Bottom-Up	Venom Pre-Fractionation	Digest	Post-Fractionation	Data Analysis Type	Database	Identified Toxins	Reference
*Parasteatoda tepidariorum* * (Theridiidae)		X	SDS-PAGE (8 fractions)	in-gel trypsin	online RP-HPLC	db-dependend	predicted transcripts from genome assembly	≥99 distinct proteins	[23]
*Cupiennius salei* * (Trechaleidae)		X	SEC, desalting	trypsin, chymotrypsin	online RP-HPLC	db-dependend	UniProtKB Araneae sequences + identified transcripts	49 peptides, ≥23 proteins	[107]
	X		SEC, desalting, online RP-HPLC	-	-	db-dependend			
*Tetragnatha versicolor* * (Tetragnathidae)		X	SDS-PAGE (3 fractions)	in-gel trypsin	NA, (probably online RP-HPLC)	db-dependend	translated transcriptome sequences + NCBI chelicerate sequences	62 distinct proteins in 31 clusters	[35]
*Phoneutria nigriventer* * (Ctenidae)		X	-	trypsin	MudPIT	db-dependend	predicted proteins from transcriptome	29 cys-rich peptide toxins	[154]
*Selenocosmia crassipes* (sub *Phlogius crassipes*) (Theraphosidae)		X	-	trypsin	online RP-HPLC	db-dependend	UniProtKB Arthropoda sequences	Ontogenetic analysis, depending on group: 12, 11, 8, or 8	[265]
		X	SDS-PAGE	in-gel trypsin	online RP-HPLC				
*Pamphobeteus verdolaga* * (Theraphosidae)		X	RP-HPLC	Lys-C/trypsin	online RP-HPLC	db-dependend	some with sequences from UniProt, NCBI or the ArachnoServer	16 peptides	[269]
*Loxosceles intermedia* (Sicariidae)		X	10 kDa cutoff spin-column	trypsin, pepsin, chymotrypsin	online RP-HPLC/ MudPIT	de-novo, spectral-network algorithmes	*de-novo* sequences against *Loxosceles* UniProt sequences + *L. intermedia* vg transcriptome	190 proteins	[266]
*Poecilotheria Formosa* * (Theraphosidae) *Viridasius fasciatus* * (Viridasiidae) *Latrodectus mactans* * (Theridiidae) *Heteropoda davidbowie* * (Sparassidae)		X	solid phase extraction	NA	online RP-HPLC	db-dependend	assembled contigs from transcriptome sequencing	103 9 4 68	[225]
*Plectreurys tristis* * (Plectreuridae)		X	SDS-PAGE	in-gel trypsin	-	db-dependend	plectreurid transcriptome, private haplogyne vg cDNA libraries, chelicerate seq. in NCBI	7 astacins-like groups, 7 peptide groups, 1 unknown	[230]
*Latrodectus Hesperus* * (Theridiidae)		X	NA	trypsin	MudPIT	db-dependend	*Latrodectus hesperus* seq. on NCBI, ESTs (vg specific) + assembled transcriptome	61	[22]
*Trittame loki* * (Barychelidae)		X	NA	trypsin	online RP-HPLC	db-dependend	translated cDNA library	45	[21]
	X		online RP-HPLC	-	-				
*Araneus ventricosus* * (Araneidae)		X	2D gel electrophoresis (106 spots)	in-gel trypsin	online RP-HPLC	db-dependend, de-novo	NCBInr with animal species restriction, de-novo sequences matched against transcriptome	db-dependend (functional analysis): 65 de-novo: 130	[236]
		X	-	trypsin	online RP-HPLC				
*Latrodectus tredecimguttatus* (Theridiidae)		X	SDS-PAGE	in-gel trypsin	online RP-HPLC	db-dependend, de-novo	NCBInr with animal species restriction	75 venom proteins	[270]
		X	-	trypsin	online RP-HPLC				
*Chilobrachys guangxiensis* (sub *Chilobrachys jingzhao*) (Theraphosidae)		X	SEC, > 10 kDa: 2D gel electrophoreses,	in-gel trypsin	online RP-HPLC, -	db-dependend manually de-novo	raw genome data of the arthropod; de-novo: MS-BLAST	47 from in-gel digestion	[264]

Data not available from the cited reference is designated as NA (not available). To following abbreviations are used: SDS-PAGE = sodium dodecyl sulphate polyacrylamide gel electrophoresis, RP-HPLC = reverse phase high performance liquid chromatography, SEC = size exclusion chromatography, db = database, MudPIT = multi-dimensional protein identification technology, vg = venom gland. * Studies include transcriptomics experiments.
